# Sex Differences in Fecal Microbiome Composition and Function of Dromedary Camels in Saudi Arabia

**DOI:** 10.3390/ani12233430

**Published:** 2022-12-05

**Authors:** Haitham Elbir, Naser Abdullah Alhumam

**Affiliations:** 1 Camel Research Center, King Faisal University, P.O. Box 400, Al-Hasa 31982, Saudi Arabia; 2 Department of Microbiology, College of Veterinary Medicine, King Faisal University, P.O. Box 400, Al-Hasa 31982, Saudi Arabia

**Keywords:** dromedary camel, methane emissions, 16S rRNA gene, sex impact, bacteriome

## Abstract

**Simple Summary:**

The gastrointestinal microbiome plays a significant role in diet digestion and the energy production of its host. This study investigated the influence of sex on the fecal microbiome of camels. Our findings revealed sex differences in the composition and function of fecal microbiomes. This research, to the best of our knowledge, is the first characterization of the fecal core bacteriomes of male and female dromedary camels. The variation in the intestinal microbial communities between male and female dromedary camels lies in the abundance and prevalence of taxa rather than in the presence and absence of bacterial taxa.

**Abstract:**

The gastrointestinal microbiome plays a significant role in diet digestion and the energy production of its host. Several factors that affect the gastrointestinal microbiota composition were studied in camels. Yet, the impact of sex on the gastrointestinal bacteriome of camels remains unexplored to date. In this perspective, the fecal microbiome community composition from dromedary camels was determined in 10 male and 10 female samples using the 16S rRNA amplicon, in order to estimate if this was influenced by sex. The core microbiome in females contained 284 bacterial OTUs and one archaeal OUT, whereas in males, it contained 279 bacterial OTUs and one archaeal OTU. In females, Bacteroidetes and Spirochaetes were significantly more abundant than in male camels, whereas Lentisphaerae and Euryarchaeota were significantly abundant in males. According to Principal Coordinate Analysis and UPGMA clustering, grouping with respect to sex was observed. The functional prediction results showed differences such as energy production and conversion, and that the cell wall/membrane/envelope were enriched in female camels. The fecal microbiome of male camels was rich in amino acid, lipid transport and metabolism.

## 1. Introduction

An animal’s gut microbiome is shaped by several factors such as diet and age [1]. Sex, in particular, is a neglected factor in several studies. Nonetheless, in studies that consider sex as a factor, they found contradicting results, as one study found a limited effect on gut microbiota [2] while other studies have suggested evidence for sex impact on gut microbiota [3,4]. Furthermore, two previous studies revealed that the commensal microbial community can impact sex hormone levels [3,4]. A recent study revealed that Bacteroidetes was more abundant in fecal microorganisms of female pigs than in male pigs. In contrast, Firmicutes was higher in male pigs than in female pigs and further research revealed that castration can affect the composition of the gut microbial community [5]. Another study in Tibetan goats showed that Fibrobacteres and Spirochaetes had greater relative abundances in the microbiota of females than in males [6]. All together, these studies indicate that sex represents an important factor in shaping the gut microbial community in animals.

Camels have unique characteristics in physiology, biochemistry and morphology, which allows them to withstand hot climates and water and food shortages in the desert [7]. Unlike a true ruminant forestomach that has four compartments, camels have pseudo-ruminants, with their forestomach consisting of three compartments and lacking an omasum [8]. We think these unique characteristics of camels would lead to distinctive gastrointestinal bacteriome that deserve to be explored. Nevertheless, previous studies focused only on the bacterial community in the gastrointestinal tract of camels [9,10,11,12,13] and the effect of age and dietary alterations on microbiome composition [14,15,16,17,18,19,20,21]. 

A gut’s microbiome can influence the host immunity, which indirectly mirrors the level of disease resistance due to sex variation [22]. Furthermore, studying the sex impact on the microbiome might clarify if sex is a statistical variable or not. To our knowledge, no published information on whether the fecal microbiomes of dromedary camels were different between the adult male and adult female camels is available. Thus, this study aimed to extend the previous findings of other animals by investigating any significant sex differences at the OTUs level. Therefore, we explore sex differences in the composition and function of fecal microbiomes in dromedary camels using the 16S rRNA amplicon.

## 2. Material and Methods

### 2.1. Samples Collection 

Fecal samples were collected on April 2021 from 10 male camels 10 female camels with an average age of 6 years old. All dromedary camels involved in this study were part of a camel herd registered with the Camel Research Center farm, King Faisal University, which is located in Al-Hasa province, Saudi Arabia. All the camels were healthy and did not receive any medication. All camels were raised in the same farm conditions, consumed the same diet and clean water ad libitum. Each camel was offered a daily diet that consisted of 2 kg alfalfa forage at 06:00 h and 2 kg feed concentrate at 15:00 h. The feed concentrate consisted of wheat bran, barley, corn, soybean meal and molasses. The chemical composition of the feed concentrate was 18% protein, 3% crude fat, 6% crude fiber, 6.5% ash, 0.7%NaCl, 0.6%phosphorous, 1% potassium, 1% calcium, 0.23% magnesium, 3 IU/g vitamin D, 20 IU/g vitamin A, 15 IU/kg vitamin E and 2780 kcl/kg metabolizable energy. 

Feces were collected from each camel into falcon™ 50 mL conical centrifuge tubes. The collected fecal samples were stored at −20 °C, before whole DNA was extracted using the QIAamp Fast DNA Stool Mini Kit (Qiagen, Hilden, Germany), according to the manufacturer’s instructions and kept at −20 °C until they were used as a template for sequencing. 

### 2.2. V3-V4 16S rRNA Amplicon Sequencing and Bioinformatics Analysis

Briefly, the V3-V4 region of the 16S rRNA gene was amplified with Bakt_341F and Bakt_805R primers [23]. The amplicon library was prepared by ligating sequencing adapters and indices to purified PCR products using the Nextera XT DNA library Kit (Illumina, San Diego, CA, USA) according to the 16S rRNA metataxonomics sequencing library preparation protocol (Illumina, San Diego, CA, USA). Then, the libraries’ concentrations was determined, and equimolar volumes of each of the libraries was pooled and processed on an Illumina’s Miseq platform with paired-end 300 bp reads by Macrogen Inc (Seoul, Republic of Korea). MiSeq reads were assembled by FLASH version 1.2.11 [24], which merge overlapping paired-end reads. Read trimming, filtering with a quality score offset 33 and out selection with a 97% identity cut-off was done using CD-HoutOTU software [25]. OTUs were classified by using the RDP Ribosomal Database Project11.5 classifier [26] and by a blast against the NCBI 16S rRNA database with BLASTN using default parameters [27]. For species-level identification using V3-V4 16S rRNA sequences region, Villmones et al., 2018 [28], recommends ≥99.3% similarity with a trusted reference species together with a minimum distance of >0.8% to the closest species. Based on the levels of intra-species sequence variations, we observed them in Genbank sequences, and adopted a more stringent cut off ≥1% minimum distance to the closest species while keeping a similarity of ≥99.3%.

QIIME software was used to perform rarefaction curves and alpha diversity analyses (Chao1 index and sample coverage) [29]. For beta diversity statistical analysis, we used the diversity plugin from QIIME. An unweighted unifrac distance matrix [30] was constructed from the phylogenetic tree and visualized using principal coordinates analysis. Hierarchical clustering of samples was constructed using the unweighted pair-group method with arithmetic mean (UPGMA).

We used PICRUSt2 to predict the functional gene content of bacteria [31] by using homology for genes in the COG database [32]. The significant differentially abundant OTUs between male camel and female camel were compared by STAMP [33].

## 3. Results

### 3.1. 16S rRNA Sequence Analysis and OTUs Clustering 

For 20 fecal samples, after removal, we obtained low quality and chimeric reads, for a total of 332,442 high quality reads. OTUs for each sample were identified at a 97% sequence similarity level. A total of 1673 OTUs were detected in the 20 camels located at the university farm. On average, the number of OTUs was higher in females than in males but this difference was not significant (*p* > 0.05). According to the 0.99% average good’s coverage estimate, the sequencing depth was sufficient to estimate 99% of the bacterial diversity and species richness in all samples (Table 1). An alpha diversity analysis (Table 1) showed higher values for females than for males regarding the Chao1 index, but this difference was not significant (*p* > 0.05), whereas the Shannon index was higher for males than for females, although this difference was also not significant (*p* > 0.05). As for the Gini–Simpson index, the values were similar and, therefore, were not significant (*p* > 0.05).

### 3.2. Prevalence Rate and Abundance of Bacteria/Archaea in Feces 

**Figure 1:**
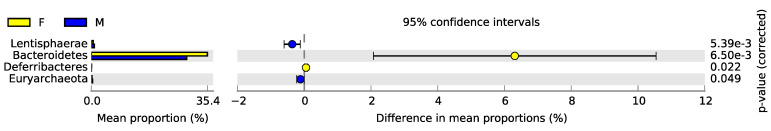
Extended error bar plot indicating the significant differences in mean abundance of phyla between males and females.

At the herd level, a total of 1473 OTUs (88.05%) out of 1673 OTUs were assigned to 16 bacterial phyla and 2 archaea, whereas 11.95% of OTUs were unclassifiable (Table 2). As for bacteria, Firmicutes, Bacteroidetes, Verrucomicrobia, Proteobacteria, Spirochaetes, Fusobacteria, Lentisphaerae and Euryarchaeota were dominant in all samples. The proportion of these eight phyla was 88.8% in entire female camels and 89% in male camels. As for the archaea, two OTUs were assigned to the phylum Candidatus Thermoplasmatota and three OTUs were assigned to Euryarchaeota. OTU1072 and OTU542 were assigned to the genus *Methanobrevibacter,* whereas OTU22 was assigned to the genus *Methanocorpusculum*. Firmicutes and Bacteroidetes constituted the two abundant phyla of both groups (Table 2). In addition, Bacteroidetes had significantly greater relative abundance in females than in male camels (*p* < 0.05). In contrast, Lentisphaerae and Euryarchaeota were significantly more abundant in male than in female camels (*p* < 0.05). As for the archaea phylum, Euryarchaeota was significantly more abundant in male than female camels (*p* < 0.05) (Figure 1).

At the OTUs level, we grouped the 1673 OTUs detected in camels located at the university farm into high prevalent (core), moderate and low prevalent bacteria/archaea groups depending on their prevalence. The core bacteriome of female camels consisted of 284 OTUs representing 87.1% of the total reads. In contrast, the core bacteriome of male camels consisted of 279 OTUs representing 82.8% of the total reads. We classified the OTUs with a prevalence of less than 100% to 50% into the moderate prevalent bacteria group, which accounted for 10.8% and 14.8% of the total reads in the females and males, respectively. OTUs with a prevalence of less than 50% were classified into the low prevalent bacteria group. They accounted for 2.1% and 2.4% of the total reads in the females and males, respectively. The female core is dominated by Firmicutes, at 33.1% followed by Bacteroidetes at 22.5%, Verrucomicrobia at 15.1% and unclassified bacteria at 8.3%. The the male core is dominated by Firmicutes at 33.5%, followed by Bacteroidetes at 23.3%, unclassified bacteria at 8.7% and Verrucomicrobia at 7.4%. Out of the 16 phyla, 10 and 9 phyla were present in the core of females and males, respectively. As for the archaea, only one OTU was represented in the core from the genus *Methanocorpusculum* in male and female camels.

**Figure 2:**
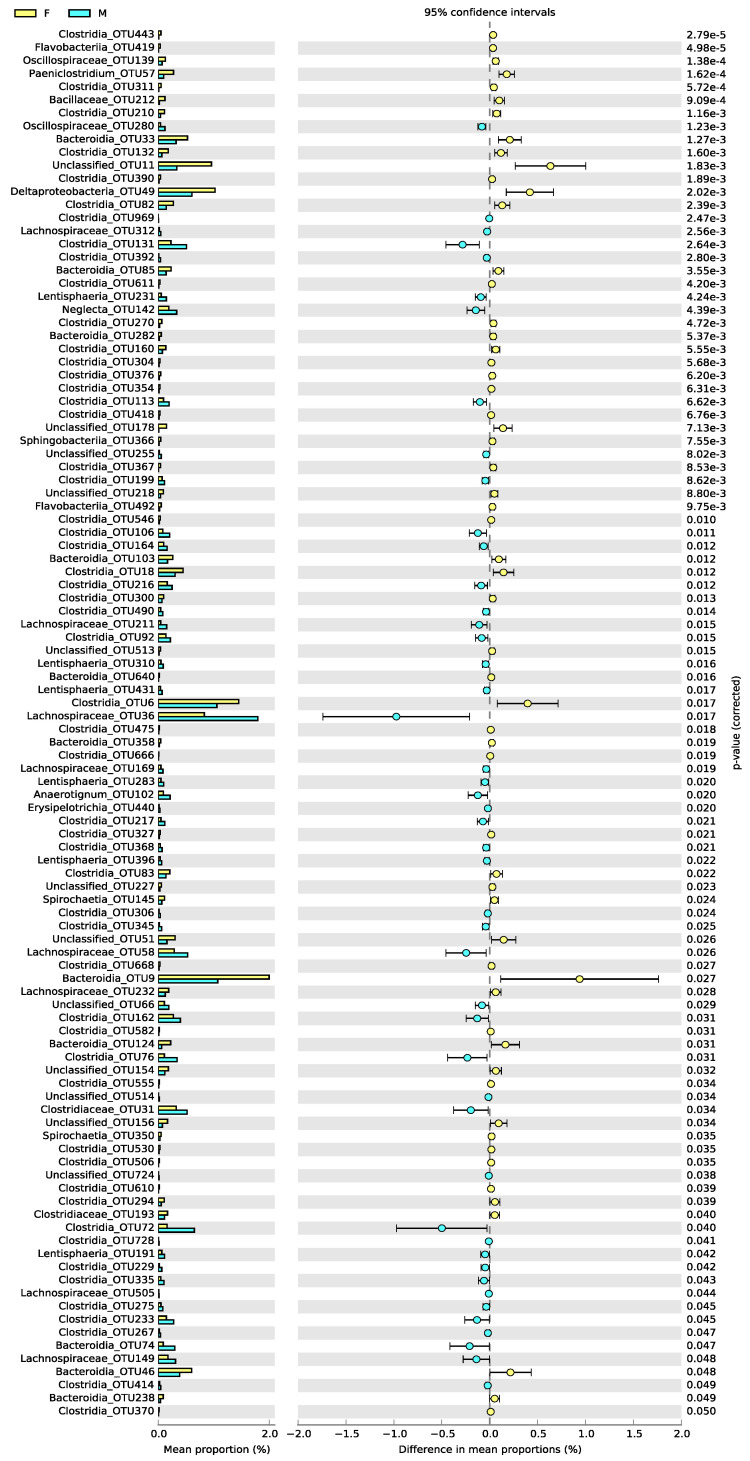
Extended error bar plot indicating the significant differences in mean abundance of OTUs between males and females.

By analyzing the differences of OTUs’ abundance in females and males, we detected a total of 106 OTUs with statistical differences between male and female camels. Of these, fifty-nine OTUs were significantly more abundant in the core of female camels than in the core of male camels. On the contrary, forty-seven OTUs in the cores of male camels were significantly more abundant than in the core of female camels (Figure 2). The significant OTUs in the core of male camels were distributed in Firmicutes (76.6%), Lentisphaerae (12.8%), Bacteroidetes (2.1%) and unclassified (8.5%). As for the females, the significant OTUs in the cores of female camels were distributed in Firmicutes (59.3%), Bacteroidetes (22%), unclassified (13.6%), Proteobacteria (1.7%) and Spirochaetes (3.4%). Data also showed that the prevalence of *Succinivibrio dextrinosolvens* in male camels is found in the low prevalent bacteria group whereas it is a core member in the female camels.

### 3.3. Comparison of Microbial Community

**Figure 3:**
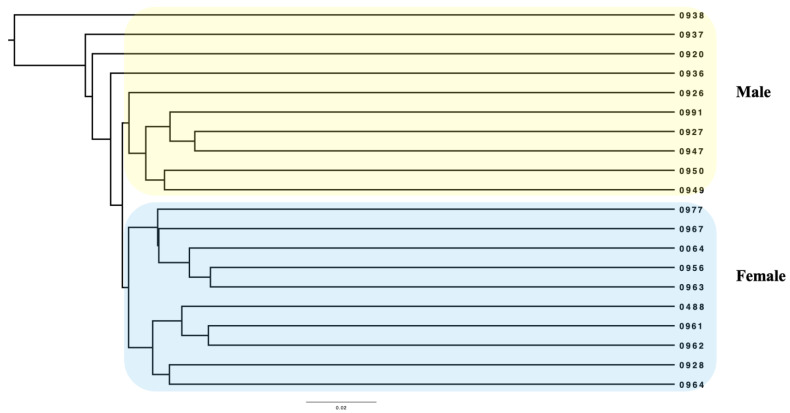
Dendrogram generated by UPGMA clustering analysis.

For comparing microbial profiles between male and female camels, we applied the unweighted UniFrac phylogenetic distance matrixes approach, which considers the presence/absence of species by weighing the relative abundances. The unweighted pair-group method with arithmetic mean (UPGMA) method is a type of hierarchical clustering that is used in the ecology of group samples. Based on the unweighted Unifrac dissimilarity, the UPGMA cluster tree revealed that the microbiomes of female camels were clustered in one group, whereas most of the microbiomes of male camels were clustered in another group (Figure 3).

**Figure 4:**
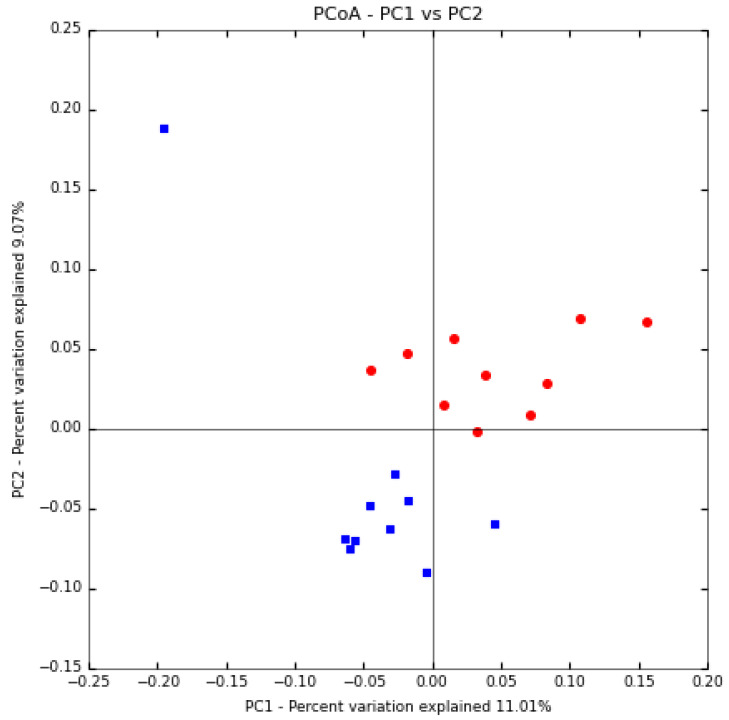
Principal coordinate analysis. Blue dots are for male samples and red dots for females.

Principal Coordinate Analysis (PCoA) is an approach that helps with extracting and visualizing highly informative components of variation of data. The unweighted UniFrac coefficients are calculated to assist in the PCoA analysis, and the result is shown in (Figure 4). The male and female samples were distributed in different locations, demonstrating the significant variation between the male and female camels, except for one sample from the males. Therefore, the PCoA showed clusters according to sex.

### 3.4. Differences of Microbial Function among Male and Female Camels

COG is a known protein functional classification database for prokaryotes. Utilizing this, a total of 25 metabolic pathways was studied. Among the principal COG gene families (Figure S1), five COG gene families with functions significantly more abundant in female than male camels (*p* < 0.05) include: energy production and conversion, posttranslational modification, protein turnover, chaperones, cell wall/membrane/envelope biogenesis, inorganic ion transport, metabolism and secondary metabolites biosynthesis, transport and catabolism, which were significantly more abundant in female than male camels (*p* < 0.05). As for male camels, functions related to transcription, replication, recombination and repair, cell cycle control, cell division, chromosome partitioning, signal transduction mechanisms, cell motility, extracellular structures, carbohydrate transport and metabolism, amino acid transport and metabolism, nucleotide transport and metabolism, coenzyme transport and metabolism and lipid transport and metabolism were significantly higher in male camels than in female camels (*p* < 0.05).

## 4. Discussion

This study unveils the sex differences in the abundance and function of intestinal bacteriome of the dromedary camel. It has been published that sex differences lead to differences in the gastrointestinal microbiomes of animals [4]. The gut microbiomes of animals can be influenced by several factors. Of these factors, diet plays a major role over other factors such as age, geographical location and environment in shaping the animal’s gastrointestinal microbiota [34]. In our study, we ensured the homogenization of factors such as diet, environment and geographical location. As for age, we included adult camels as their rumen microbial diversity is relatively stable when ruminants approach adulthood [35]. As sex differences in gut microbiota are not visible until puberty, the impact of sex hormones in shaping gastrointestinal microbiota composition is supported [3,4]. Despite the homogenization of confounding variables, significant differences could be detected in bacteriome composition and functionality related to sex. Thus, data reported herein were interpreted as authentic.

The large intestine (colon, cecum and rectum) serves as a second site for fermentation of undigested nutrients that escape fermentation and absorption in the rumen. Therefore, the large intestine enhances the overall energy extracted from a diet. In addition, it has been associated with production traits, in particular with regards to the efficiency of milk production in cows [36].

The idea of camel core fecal bacteriome is still evolving. A number of previous studies described the core rumen bacteriome in camels [11,16]. However, the lack of consistency regarding criteria for a core definition may lead to incomparable data. This study, to the best of our knowledge, is the first description of the fecal core bacteriomes of male (279 OTUs) and female (284 OTUs) dromedary camels. In this study, the low prevalence and abundance of some bacteria suggest they are likely transient bacteria acquired from the surrounding environment.

Beta analysis revealed that there were some differences in the content of fecal bacteriomes between the two groups. Intestinal microorganisms are influenced by host hormones [4]. In this research, PCoA and UPGMA analysis showed that male camel samples were not clustered with female camel samples except for one male sample clustered within the female samples. One explanation is that the male samples have different levels of sex hormones concentration. One of the limitations of our study is that the sex hormone level was not measured.

Form the physiological point of view, adult female camels are unlike male camels in that they need more energy to meet the energy requirement for the lactation and reproduction process. To fulfil their energy requirements, camels rely on rumen microbiomes, which ferment plant material into metabolic end products such as volatile fatty acids (VFAs) and methane. VFAs are absorbed by the rumen wall and act as an energy source for animal [37,38]. As for methane, it is not absorbed but released into the atmosphere together with its retained energy, thus contributing to energy loss from the feed [39]. However, female camels used in our study were not lactating and may have experienced similar stress as the male camels. Therefore, different sex hormones levels between males and females might be one of the possible explanations for this difference. Researchers have found that sex hormones such as estrogen and testosterone directly shape the gut microbiome [40]. The cross-talk between microbiota and sex hormones likely acts by directly affecting the growth of specific taxa and by influencing the immune response to gut microbiota [40]. Anyhow, the whole mechanism through which hormones affect the host’s selection of gut microbial communities is currently not fully understood.

The present study sequenced the fecal bacteriomes in dromedary camel of different sexes and found that the prevalence and abundance of bacteriomes were significantly different between males and females. Previous studies of camels revealed that Firmicutes and Bacteroidetes harbour many genes encoding carbohydrate active enzymes, thereby assisting the host’s breakdowns and fermenting of dietary carbohydrates [16]. Here, we found most significant abundant OTUs (biomarkers) in male camels were found in Firmicutes and Lentisphaerae, whereas the potential biomarkers of female camels were mainly found in Firmicutes and Bacteroidetes, which is in line with the findings of Wang et al. [5]. Another two potential biomarkers in female camels were distributed in Spirochaetes, which contained genes encoding enzymes for the hydrolysis of cellulose and pectin [41]. Herein, we found that the relative abundance of Spirochaetes in female camels was significantly higher than that in male camels, which is consistent with the results found in female Tibetan goats [6]. The asaccharolytic genus *Anaerotignum* is significantly abundant in male than female camels. The Oscillospiraceae species, which are cellulolytic, are significantly more abundant in females than in male camels. Flavobacteriia are significantly more abundant in females than in male camels. Of the other potential biomarkers identified, *Succinivibrio dextrinosolvens* was more abundant in females than in males. Notably, *S. dextrinosolvens*, a species in Succinivibrionaceae, was isolated before from the cattle rumen and was abundant in high-yielding multiparous cows. Hailemariam et al. showed that *S. dextrinosolvens* Z6 plays a role in nitrogen utilization [42].

The archaeal domain in the feces largely consists of methanogenic archaea from the phylum Euryarchaeota. These methanogens are involved in methane production, which in turn is eructed and released into the atmosphere. *Methanobrevibacter*, a member of Euryarchaeota, is the prevalent genus of the archaea community, constituting more than 70% of the total archaea and is the major contributor to methane production in ruminant rumen [1]. Unlike the previous studies, *Methanobrevibacter* spp. was not dominant, but instead *Methanocorpusculum* was a core member and showed the highest relative abundance in the fecal archaeal community of camels. During the digestion process of plant material, methane is produced by methanogenic archaea and is not absorbed by rumen but released into the atmosphere together with its retained energy [39], thus contributing to energy loss from the feed. In this study, the higher abundance of methanogenic archaea in male camels than female camels warrants further analysis on its impact on energy waste by methane release.

Fecal bacteriomes contain around 672,015 genes. This microecosystem executes different functions when the content of gastrointestinal microbial community is altered. To analyze functional differences among male/female camels, we used the groups species content information to predict the functional gene content. The functional differences between the fecal bacteriomes of male camels and female camels was significant. In the COG gene family, energy production and conversion and cell wall/membrane/envelope were significantly increased in the female group. In contrast, males showed enrichment in COG pathways related to amino acid transport and metabolism and carbohydrate transport and metabolism. A related study exploring sex differences in the composition and function of intestinal microorganisms in pigs found a similar enrichment in the same COG pathways mentioned above [5]. Collectively, these differences further confirm that the sex factor has an impact on the fecal bacteriome composition.

## 5. Conclusions

This study provides preliminary information on the potential relationship of camel fecal microbiome composition and host sex. The differences in the intestinal microbial communities between male and female camels lies in the abundance and prevalence of taxa rather than in the presence and absence of taxa. Thus, sex should be taken into consideration in future research of fecal microbiomes in camels.

## Figures and Tables

**Table 1:** Number of samples analyzed, estimated OTU richness (Chao1), Shannon index, GiniSimpson index and estimated sample coverage for 16S rRNA libraries. Sample level composition of OTUs.

Sample Name	Group	Age	OTUs	Chao1	Shannon	Gini–Simpson	Good’s Coverage
64	Female	3	715	818.511	7.359	0.983	0.991
488	Female	10	679	788.879	6.418	0.932	0.991
928	Female	9	730	864.639	7.592	0.988	0.991
961	Female	11	674	813.276	6.436	0.938	0.991
962	Female	11	587	769.043	5.639	0.907	0.990
963	Female	7	664	770.139	6.940	0.968	0.992
964	Female	3	749	861.258	6.445	0.931	0.993
967	Female	4	709	823.079	6.671	0.947	0.990
977	Female	3	707	864.120	6.373	0.941	0.991
998	Female	3	713	782.515	7.426	0.979	0.992
920	Male	3	703	790.073	7.069	0.981	0.995
926	Male	4	719	821.070	6.364	0.931	0.993
927	Male	3	700	783.875	7.344	0.979	0.993
936	Male	7	670	747.351	6.531	0.947	0.993
937	Male	10	627	710.023	5.963	0.916	0.993
938	Male	3	637	747.915	7.035	0.973	0.993
947	Male	3	731	818.079	7.565	0.987	0.993
949	Male	12	700	826.110	7.192	0.977	0.991
950	Male	4	728	808.010	7.558	0.987	0.993
991	Male	6	659	759.309	6.872	0.961	0.988

**Table 2:** Average relative abundance of bacteria and archaea phyla found in fecal samples and phylum level composition of OTUs. Values were presented as %.

Kingdom	Phylum	Number of OTUs		Average Relative Abundance	
		Male	Female	Male	Female
Bacteria	Firmicutes	769	990	41.315	36.146
Unclassified	Unclassified	83	165	10.092	10.868
Bacteria	Bacteroidetes	140	197	26.105	31.538
Bacteria	Proteobacteria	38	45	5.705	4.85
Bacteria	Spirochaetes	18	23	2.371	2.317
Bacteria	Lentisphaerae	21	22	0.796	0.47
Bacteria	Verrucomicrobia	10	16	7.627	11.366
Bacteria	Candidatus Melainabacteria	8	11	0.07	0.092
Bacteria	Actinobacteria	4	6	0.009	0.012
Bacteria	Fusobacteria	1	2	4.899	1.369
Bacteria	Planctomycetes	5	6	0.123	0.142
Bacteria	Tenericutes	5	5	0.022	0.008
Bacteria	Fibrobacteres	2	2	0.528	0.53
Bacteria	Elusimicrobia	2	2	0.025	0.041
Archaea	Euryarchaeota	3	3	0.276	0.176
Bacteria	Synergistetes	1	2	0.003	0.003
Archaea	Candidatus Thermoplasmatota	2	2	0.012	0.005
Bacteria	Chloroflexi	1	0	0.002	0
Bacteria	Deferribacteres	1	1	0.02	0.067

## Supplementary Materials

The following supporting information can be downloaded at: https://www.mdpi.com/article/10.3390/ani12233430/s1, Figure S1: COG categories were predicted by PICRUSt2.
