# Supplementary material for: Sex Differences in Fecal Microbiome Composition and Function of Dromedary Camels in Saudi Arabia
Source: Animals (Basel). 2022 Dec 5;12(23):3430. doi: 10.3390/ani12233430 (PMC9736497; doi:10.3390/ani12233430)
Supplement: Supplementary file 1 [file animals-1947937-supplementary.pdf › animals-1947937-supplementary.pdf]

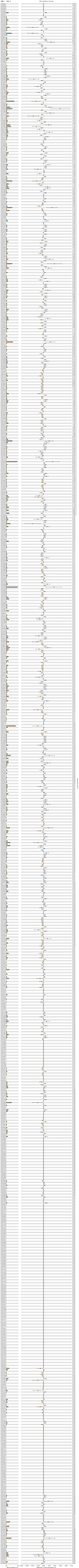

Figure S1. COG categories were predicted by PICRUSt2. Only the significantly different COG categories are shown
